# Treatment of Idiots

**Published:** 1848-10-01

**Authors:** 


### TREATMENT OF IDIOTS.

This subject appears to be engaging considerable attention in Massachusets, where a Commission, appointed by the Governor, reported that in 171 towns, containing 345,285 inhabitants, there were 543 idiots, (204 males and 339 females.) Of these, 169 are less than twenty-five years of age, and, of course, proper subjects for instruction. Of the whole number, 106 were supported entirely at the public charge. The whole state is estimated to contain upwards of 1000 idiots, of whom 300 are of proper age for instruction. Information, by personal inspection of the idiots, in about thirty towns in various parts of the state, showed that their condition was very materially influenced by the character of those who have the charge of them. The Committee state—

"In some towns we found the idiots, who were under the charge of kind-hearted but ignorant persons, to be entirely idle, given over to disgusting and degrading habits, and presenting the sad and demoralizing spectacle of men, made in God's image, whom neither their own reason, nor the reason of others, lifted up above the level of the brutes. In other towns, idiots, who, to all appearance, had no more capacity than those just mentioned, were under the charge of more intelligent persons, and they presented a different spectacle: they were healthy, cleanly, and industrious. We found some, of a very low grade of intellect, at work in the fields, under the direction of attendants; and they seemed not only to be free from depraving habits, but to be happy and useful. The inference to be drawn from this is very important. If persons having only common sense and common humanity, but without the advantage of experience or study, can so improve the condition of idiots, how much could be done by those who should bring the light of science, and the experience of wise and good men in other countries, and the facilities of an institution adapted to the training of idiots,—how much, we say, could be done by such persons, towards redeeming the minds of this unfortunate class from the waste and desolation in which they now lie!"

Appended to the Report is a very interesting letter from Mr. George Sumner, of Boston, Mass., written from Paris, and describing the system of instruction for idiots introduced there with great success. He writes—

"During the past six months, I have watched, with eager interest, the progress which many young idiots have made, in Paris, under the direction of M. Seguin; and at Biçetre, under that of Messrs. Voisin and Vallée; and have seen, with no less gratification than astonishment, nearly one hundred fellow-beings who, but a short time since, were shut out from all communion with mankind,—who were objects of loathing and disgust, many of whom rejected every article of clothing; others of whom, unable to stand erect, crouched themselves in corners, and gave signs of life only by piteous howls; others, in whom the faculty of speech had never been developed; and many, whose voracious and indiscriminate gluttony satisfied itself with whatever they could lay hands upon, with the garbage thrown to swine, or with their own excrements. These unfortunate beings (the rejected of humanity) I have seen properly clad, standing erect, walking, speaking, eating in an orderly manner at a common table, working quietly as carpenters and farmers; gaining, by their own labour, the means of existence; storing their awakened intelligence by reading one to another; exercising towards their teachers and among themselves, the generous feeling of man's nature; and singing, in unison, songs of thanksgiving!"

In 1828, Dr. Ferries, then the principal physician at Biçetre, undertook the education of a few of the more intelligent idiots; and in 1839, when

Dr. Voisin was made physician of that establishment, a school was organized, which, although producing some good results, was very incomplete, until M. Seguin,—who, since 1837, had occupied himself in private with the education of idiots,—was, in 1842, named director. He had conceived and put in practice a method of education, the happy results of which were certified to by Esquirol in 1839, and subsequently by various committees. He remained only a year at Biçetre, and the school has since been under Dr. Voisin, and under the especial direction of M. Vallée, while M. Seguin has confined himself to private instruction in Paris. In Switzerland, Dr. Guggenbühl had founded an establishment for the education of *crêtins,* which, within the past three years, has been most satisfactory in its results. More recently, in Berlin, Dr. Saegert has commenced the education of some twenty idiots, and has given the results of one year's efforts in a pamphlet.

M. Seguin, in his large work, explains the method which he has been led by experience and reflection to adopt:

"He considers their treatment and education as possible on two conditions:—First, that the treatment be not only hygienic, but moral; and, secondly, that the education be, not the putting in action of acquired faculties—which is the education of common schools—but the development of the functions, of the aptitudes, of the faculties, and of the instinctive and moral tendencies. These must be ascertained by a careful physiological and psychological examination or analysis of each case, a form or table for which is proposed by him. Another table proposed by Voisin, you will find in his memoir. The education of idiots may, of course, be attempted at any age, but little success can be counted on unless it commences when they are young; indeed, Seguin considers this success to be the exception to a rule which applies not alone to idiots—viz., that the aptitude to receive instruction is peculiar to youth. After the prior examination has been made, the education is commenced upon—1st, The moving power; and is followed up by 2nd, the senses; 3rd, the perceptive faculties; 4th, by gymnastics of comparison; 5th, by gymnastics of invention; 6th, excitement of sentiments and instincts by moral necessities; 7th, special excitation of the faculty of spontaneousness; 8th, incessant provocation to regular action, to speaking, and to the exercise of faculties then developed. The aptitudes thus created are then applied to different specialities, according to the fortune, age, or position of each individual, taking care to choose, in every case, an occupation which will keep in activity the muscular system as well as the mental faculties. M. Seguin assures me, that the average proportion of cases which have come to his knowledge, in which this treatment has failed of success, is not more than one in a hundred; and if nothing more be done, the repulsive symptoms of idiocy, which are all the result of *habit,* and not imposed by nature, may at least be removed.

"One great task in the moral education, which commences, however, with the first contact of the teacher and pupil, is to inspire the sentiment of authority, and, relative to this, the duty or faculty of obedience. Experience has shown that it is not by severity that this can be brought about, for that can seldom be long maintained; and the alternations which teachers who indulge in passionate severity so often present, of brutal harshness and insignificant weakness, inspire in the idiot, as well as in every one who thinks, the idea, *not* that the authority embodied in his teacher is the firm, calm expression of a moral law, but that it is the result of caprice and selfishness, against which his own instinct of self-defence compels him to combat. Brute force and distrust never yet created anything good; not so firmness, calmness, sympathy, justice."

What the success has been, and what the present school at Biçetre is,—thanks to the efforts of Seguin, Voisin, and Vallée,—will be best seen by describing a day's work at the school:—

"The number of pupils in the school has varied, for some time past, from 80 to 100. At five o'clock they rise, and pass half an hour in washing, combing, and dressing; the monitors, pupils more advanced, aiding those whose instruction is but recently commenced. Then they pass into the hall of classes, and range themselves in a double line (no easy task for the beginners), when they sing a simple morning prayer, repeated to them by the teacher. After this, they make their first breakfast, of a simple slice of bread. The class for the education of the senses now begins, and fills up the time till $8\frac{1}{4}$ a.m. In the first or highest division, several occupy themselves with face and landscape drawing; and others, less advanced, with geometrical drawing upon the black board. The third division, divided into sections, is of those who are exercising the senses of smell, taste, sight, and observing colour and form, by the method I have before described. The sense of hearing is exercised, among other means, by the pupils learning to distinguish and name, while blindfolded, the natural sounds as produced by the cords of a bass viol. Meanwhile, the youngest class of eighteen or twenty, is going through its elementary gymnastics of the moving power.

"From $8\frac{1}{4}$ to 9 a.m. is taken up by the study of *numeration* and *arithmetic.* Here the whole school is divided into frequently-changing groups, according to the various capacities developed. The lowest of all is ranged in line, and taught to count aloud up to 30,—a series of sticks, balls, or other material objects, being given them at the time. This helps to ameliorate their speech, and to stimulate to imitation those who have not that faculty. Another group is set to climb upon ladders, counting the number of rounds as they go up,—and thus the muscular system and knowledge of numeration are simultaneously developed. A higher group is of those who count up to 50 with counters, and who, by means of them, get an idea of unity, plurality, subtraction, addition, and equality. A higher group still has learned to count up to 100, and another group is learning, by means of moveable figures taken from a case, the combination of numbers. Higher still are boys working upon their slates, or going through calculations upon the black board, with a facility and precision that any pupil of Warren Colburn might envy.

"From 9 to $9\frac{3}{4}$. Breakfast, of soup and a plate of meat. The pupils are here seated at table, and eat with fork and spoon; the more adroit aiding those less so.

"$9\frac{3}{4}$ to $10\frac{1}{2}$. Recreation in open air,—running, playing ball, driving hoop, or cultivating a small plot of ground, the hire of which, for three months, each one may gain by a certain number of tickets of good conduct.

"$10\frac{1}{2}$ to $11\frac{1}{4}$. Reading class, in which all take part, divided, however, into various groups, as before.

"$11\frac{1}{4}$ to 12. Writing class. Here the lowest group is taught only to trace on the black board, with a ruler, the line in its four positions. The next group is taught to make upon the board the rudimental characters, making the three in each line. After this, they write on slates, and, when farther advanced, the monitor being ready to guide their hands, they write in ruled books. The highest class rules its own books, and writes alternately a page of large and fine hand.

"12 to $12\frac{1}{2}$. Gymnastics.

"$12\frac{1}{2}$ to 1. Music.

"1 to $4\frac{3}{4}$. Manual labour. In this all take part; some as shoemakers, some as carpenters, or rather cabinet makers, and some as tillers of the ground. One of the best exercises for the body, *inasmuch as it compels the idiot to walk and balance himself unaided*, is that of wheeling a barrow, charged with a weight proportionate to his strength. The most stupid may be soon taught this. Others, more intelligent, wield spade and pickaxe most energetically and profitably; but nowhere does their awakened intelligence appear more satisfactorily than in the workshop of the cabinet-maker. When one of them has sawed through a plank, or nailed together two pieces of wood, or made a box, his smile of satisfaction—the consequence of 'something attempted, something done,' the real result of which he can estimate—is beautiful to see. Nor is their work by any means to be despised. With one cabinet-maker, as teacher and monitor, they performed, last year, all the work necessary for their schoolroom and dormitories, as well as for a good part of the great establishment at Biçetre. At shoemaking they show intelligence; but this is too sedentary an occupation for them. Some, however, who have quitted the school, work at it; but the greater number of them become farmers and gardeners.

"After this manual labour they dine, and after dinner play till $6\frac{1}{4}$ p.m.

"From 6¼ to 7. Grammar class; the lowest group is taught to articulate syllables; the highest, as much as in any grammar school.

"From 7 to 8¼ is passed in reading to one another, or in conversations and explanations with the teacher, upon things which may excite the reflective power; two evenings in the week, this hour is devoted to a concert and a dance.

"After this comes the evening prayer, sung by all; and then, fatigued, but happy, they retire to rest.

"Such is a day at the school of Biçetre. Every Thursday morning, the teacher takes them to walk in the country, and then inculcates elementary notions of botany, designating by their names, and impressing by their smell, taste, and sight, the qualities of different flowers and useful vegetables which they see. At the same time he explains, by locality, the first elements of geography. On Saturday evening, there is a distribution of tickets of good conduct, three of which, I have before observed, pay the rent of a garden; and one of which may buy off for another, with the consent of the teacher, the punishment adjudged for certain slight acts of negligence. You will see at once the effect which this must have upon the generous sentiments of the pupils. The sentiment of possession is developed,—the rights of property taught; but its duties and its true pleasures are at the same time impressed.

"These tickets of good conduct are given also to those who are designated, *by the pupils themselves*, as having done some kind and generous action,—as having been seen to run to the aid of one who has stumbled at play,—who had divided among his companions the *bons-bons* he may have received from a visitor,—or who had helped, in any way, one weaker than himself. Thus they are constantly on the look-out for good actions in one another; but they are most positively forbidden to report the negligences or unkind conduct which they may observe. The surveillance of the monitors is sufficient to detect these; and even were it not, M. Vallée prefers that they should go unpunished rather than that they should serve to cherish the grovelling sentiments of envy and malice, which lurk in the breast of the informer and the scandal-monger. I know no spectacle more touching than this Saturday evening distribution of the rewards of real merit."

Enough has been quoted to show that "There is nothing either visionary or impracticable in the attempt to raise this unfortunate class of our fellow-beings from the state of misery and degradation to which they have been hitherto condemned, in all ages, and in nearly every land."

